# Professor Hall, of Baltimore

**Published:** 1844-06

**Authors:** 


### PROFESSOR HALL, OF BALTIMORE.

Some months ago, Prof. Hall was arraigned before the Regents of the University of Maryland on the ground of incompetency. The graduates in March adopted the most complimentary resolutions to the professor, for his industry, talents and learning, declaring that they "listened with pleasure and improvement to his able and complete course of lectures." Y.

